# Modern Innovative Solutions to Improve Outcomes in Asthma, Breathlessness, and Chronic Obstructive Pulmonary Disease (MISSION ABC): Protocol for a Mixed-Methods Study

**DOI:** 10.2196/resprot.9228

**Published:** 2019-03-18

**Authors:** Eleanor Lanning, Emily Heiden, Jayne Longstaff, Carole Fogg, Thomas Brown, Hitasha Rupani, Ann Dewey, Daniel Neville, Thomas Jones, Ruth DeVos, Mark Mottershaw, Paul Bassett, Anoop J Chauhan

**Affiliations:** 1 Research and Innovation Portsmouth Hospitals NHS Trust Portsmouth United Kingdom; 2 School of Health Sciences and Social Work University of Portsmouth Portsmouth United Kingdom; 3 Stats Consultancy Amersham United Kingdom

**Keywords:** asthma, breathlessness, COPD, diagnosis, integration, participatory action research

## Abstract

**Background:**

A high proportion of the costs for respiratory diseases are generated by a relatively small group of patients with severe disease (recognized or unrecognized) or complex problems that include multimorbidity, at-risk behaviors, and socioeconomic disadvantage. These patients often struggle to engage with the structured, proactive, care approaches for chronic disease management advocated for asthma and chronic obstructive pulmonary disease (COPD), resulting in repeated emergency use of both primary and secondary health care. An integrated approach for the management of complex patients, incorporating both specialist and primary care teams’ expertise, may be effective in improving outcomes for such high-risk patients. However, the evidence is mixed, and there is a need for evaluation of models of integrated care in routine “real-world” clinical settings.

**Objective:**

This mixed-methods protocol examines the implementation of a novel integrated care model for patients with airways disease and undifferentiated breathlessness by using both quantitative and qualitative evaluation of processes, patient and health care professional experiences, and clinical outcomes throughout the clinic cycles. It aims to establish whether Modern Innovative Solutions to Improve Outcomes in Asthma, Breathlessness, and Chronic Obstructive Pulmonary Disease (MISSION ABC), including innovative diagnostic and self-management tools, can deliver improvements in health service use and clinical outcomes for the different patient groups (asthma, breathlessness, and COPD) and compares the 12-month period prior to the first patient visit and the 6-month period following the last visit.

**Methods:**

A combination of study designs is required to evaluate all aspects of the service: participatory action research approach, involving real-time evaluation at each clinic to inform subsequent clinics; before-and-after study for patient outcomes before and after clinic attendance; and qualitative methods (interviews and focus groups).

**Results:**

The results will be compiled and published in April 2019.

**Conclusions:**

Evaluation of the clinic cycles will include consideration of qualitative data from patients, carers, and health care professionals in addition to quantitative outcomes for service implementation and patient factors. The long-term impact of the service will be evaluated using clinical and health service outcomes.

**International Registered Report Identifier (IRRID):**

DERR1-10.2196/9228

## Introduction

### The Burden of Disease

Respiratory diseases are highly prevalent and a major cause of health care utilization in Wessex, United Kingdom. The two most common chronic respiratory diseases—asthma and chronic obstructive pulmonary disease (COPD)—are underdiagnosed, are major drivers to acute care episodes, and show poor clinical outcomes compared to other conditions in many areas of the region.

More than 1 million people in the United Kingdom are diagnosed with COPD. There is still a “prevalence gap” between the expected and actual prevalence of COPD among general practitioner (GP) practices, and in 13% of the UK population aged over 35 years, COPD is undiagnosed. These “missing millions” will likely need acute care, and 15% will only be diagnosed on admission to the hospital [1]. More than 5 million people are affected by asthma in the United Kingdom, and more than 500,000 people have severe or difficult-to-control asthma, of which 70% have an allergic subtype [2]. Patients with severe, exacerbation-prone disease are more likely to be admitted to the hospital and account for the most significant utilization of health services. Both COPD and asthma are associated with increased morbidity and mortality and can lead to disabling symptoms that impact the patient’s quality of life and well-being. COPD is the fifth most common cause of death in the United Kingdom, resulting in approximately 25,000 deaths annually. It is also the second most common cause for hospital admission in the United Kingdom, and 35% of patients are readmitted within 30 days. In 2009, asthma accounted for 1,131 deaths in the United Kingdom, triggering a National Audit of Asthma Deaths. The National Review of Asthma Deaths [3] published in 2014 concluded that many areas in the diagnosis and care of patients with asthma such as access to timely and appropriate care, use of personalized action plans, and appropriate severity assessment can be improved to reduce unnecessary deaths.

The National Health Service spends £2 billion per year on the management of asthma and COPD [4]. Both conditions have direct financial costs, additional social costs through time off work and reduced productivity, and further indirect costs through reduced quality of life and well-being. The annual health care expenditure on COPD is more than £800 million (£1.3 million per 100,000 population). The treatment of severe, exacerbation-prone COPD (exacerbation is defined as an acute worsening of respiratory symptoms requiring an increase in therapy [5]) costs ten times more than that of mild disease. COPD is responsible for 24 million lost working days annually, costing the economy £2.7 billion. Nearly 80% of costs for asthma are related to the treatment of poorly controlled disease [6], which amounts to over £1 billion per annum [2] as a direct cost and £6 billion as an indirect cost to society (time off work and lost productivity). Four of the top 10 most expensive drugs covered by the National Health Service are inhaled medications for asthma and COPD.

Shortness of breath is recorded in 1% of primary care consultations [7] and 10% of the population affected by chronic breathlessness symptoms [8]. This proportion increases to one-third in the elderly, with a significant impact on the functional status and health-related quality of life [9]. Although breathlessness is a symptom of many diseases, the referral and management systems are often specific to the diagnosis. Thus, patient visits to more than one outpatient department for breathlessness may result in an onerous clinical journey and underrecognition of comorbidity [10].

### A New Model of Care

A high proportion of the costs for respiratory diseases is generated by a relatively small group of patients with severe disease (recognized or unrecognized) or complex problems that include multimorbidity, at-risk behaviors, and socioeconomic disadvantage. Such patients often struggle to engage with the structured, proactive care approach to chronic disease management advocated for asthma and COPD, resulting in repeated emergency health care use of both primary and secondary care. An integrated approach for the management of complex patients, incorporating both specialist and primary care teams’ expertise, may be effective in improving outcomes for such high-risk patients. However, the evidence is mixed, and there is a need for evaluations of models of integrated care in routine “real-world” clinical settings.

**Figure 1:**
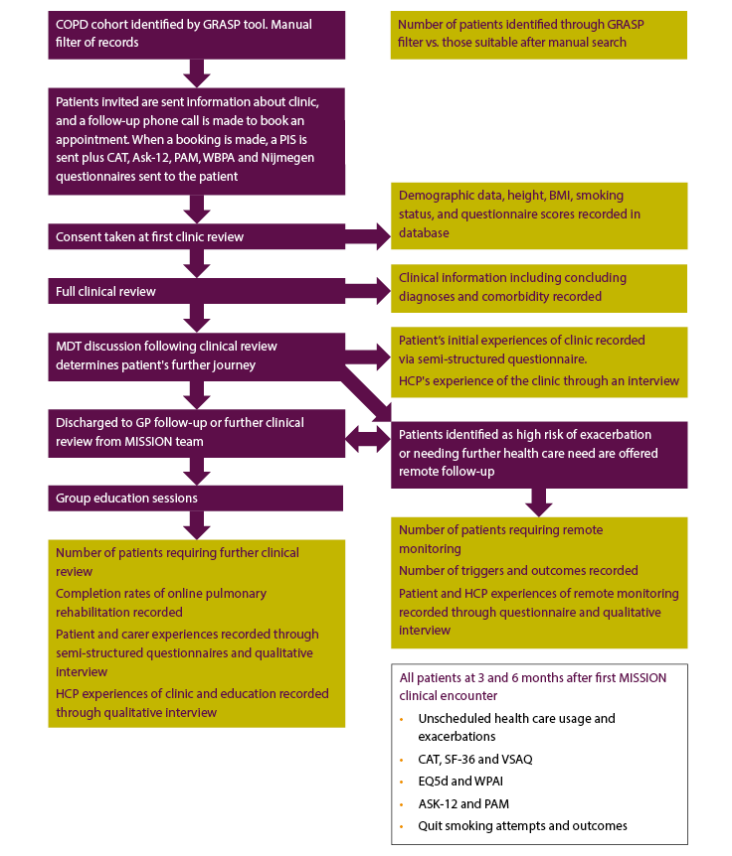
COPD cohort flow chart with study outcomes shown in yellow boxes. COPD: chronic obstructive pulmonary disease; GRASP: Guidance on Risk Assessment in Stroke Prevention; CAT: COPD Assessment Test; ASK-12: Adherence Starts with Knowledge questionnaire-12; PAM: Patient Activation Measure; WBPA: weight-bearing physical activity; MDT: multidisciplinary team; GP: general practitioner; PIS: patient information sheet; HCP: health care professional; BMI: body mass index; SF-36: Short Form Health Survey-36; VSAQ: Veterans Specific Activity Questionnaire; EQ5d: EuroQoL-5D; WPAI: Work Productivity and Activity Impairment.

**Figure 2:**
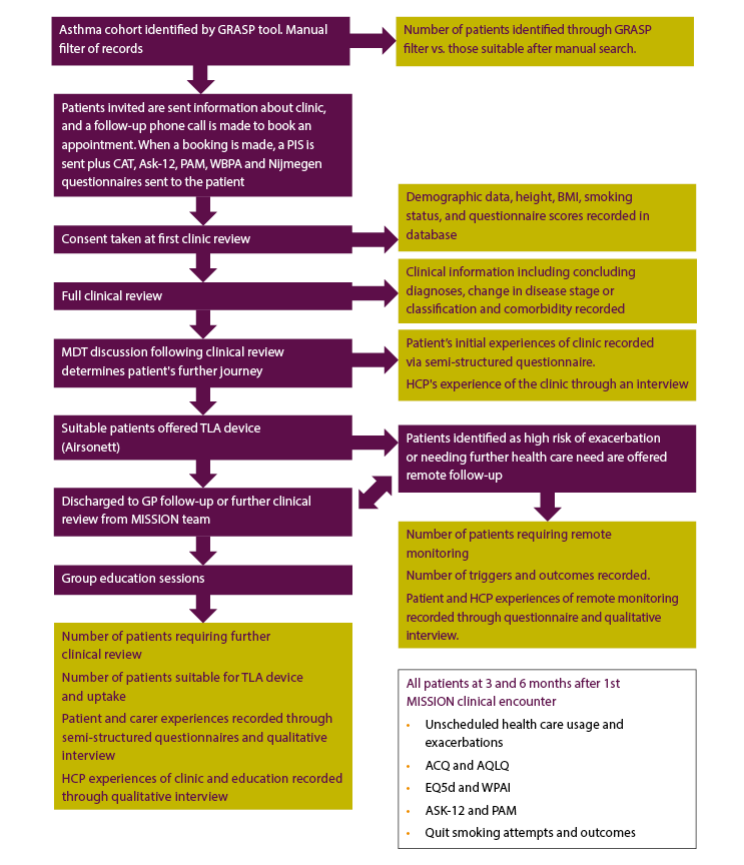
Asthma cohort flow chart with study outcomes shown in yellow boxes. GRASP: Guidance on Risk Assessment in Stroke Prevention; CAT: Chronic obstructive pulmonary disease Assessment Test; ASK-12: Adherence Starts with Knowledge questionnaire-12; PAM: Patient Activation Measure; WBPA: weight-bearing physical activity; MDT: multidisciplinary team; GP: general practitioner; PIS: patient information sheet; HCP: health care professional; BMI: body mass index; EQ5d: EuroQoL-5D; WPAI: Work Productivity and Activity Impairment; ACQ: Asthma Control Questionnaire; TLA: temperature-controlled laminar airflow.

**Figure 3:**
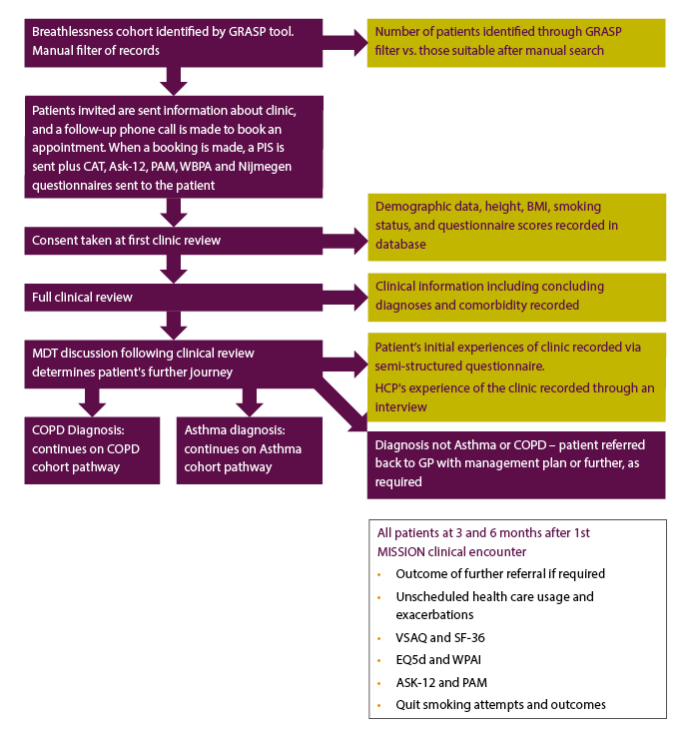
Breathlessness cohort flow chart with study outcomes shown in yellow boxes. COPD: chronic obstructive pulmonary disease; GRASP: Guidance on Risk Assessment in Stroke Prevention; CAT: COPD Assessment Test; ASK-12: Adherence Starts with Knowledge questionnaire-12; PAM: Patient Activation Measure; WBPA: weight-bearing physical activity; MDT: multidisciplinary team; PIS: patient information sheet; HCP: health care professional; BMI: body mass index; SF-36: Short Form Health Survey-36; VSAQ: Veterans Specific Activity Questionnaire; EQ5d: EuroQoL-5D; WPAI: Work Productivity and Activity Impairment.

The Modern Innovative Solutions Improving Outcomes in Asthma, Breathlessness, and COPD (MISSION ABC) system is a new model of care that starts by identifying at-risk patients and subsequently streamlines their care, incorporating new technology to improve management of airways disease. Patients are identified using criteria that indicate poor disease control, a heavy burden of symptomatology, or unidentified disease. The patients are then delivered streamlined assessment and care in a one-stop (MISSION Rapid) or two-stop clinic journey (Rapid plus MISSION Investigation clinics). MISSION Rapid clinics, organized in the community, promote integration of primary and specialist teams, wider respiratory multidisciplinary teams, psychological and well-being services, charity, and patient group representation. Each patient’s diagnosis is reviewed using spirometry, fractional exhaled nitric oxide (FeNO), and oscillometry (each provided by a specialist respiratory physiologist) along with a specialist medical review; the reasons for poor disease control are explored (unidentified comorbidity, difficulties in self-management, coexisting anxieties, or social stressors), and the medications are optimized using local and national guidelines. All patients were offered personalized self-management plans. Patients who are stable are supported in the management of their disease through education (myCOPD [11] or myAsthma [12]) and upskilling of lead health care professionals (HCPs) and champions in GP practices through competency-based training, including spirometry and comorbid disease management. The model of patient flow through the MISSION service is shown in Figure 1 (COPD), Figure 2 (asthma), and Figure 3 (breathlessness).

### A New Service for Asthma Patients

We will implement a novel clinical pathway for asthma patients that includes diagnosis, assessment, and adjustment of treatment; maintains control using complementary innovations and models of care; and draws on resources and skills from primary, secondary, and tertiary care.

We will use Guidance on Risk Assessment in Stroke Prevention (GRASP) [13] searches across Wessex Clinical Commissioning Groups to identify patients who are likely to have asthma. When a diagnosis is established, we will identify patients whose conditions are not well controlled (eg, frequent exacerbations, emergency department visits, hospital admissions, use of three or more controller medications, and use of frequent short-acting bronchodilators). These patients’ records will be reviewed by specialist nurses and, if appropriate, in MISSION clinics. The clinics will provide timely diagnosis with cutting-edge technology, including FeNO and airway oscillometry, assessment of disease control and comorbidity, an education session, and a self-management plan through written and mobile- or Web-based solutions, where appropriate. Inhaler technique and adherence will be assessed, and patients will be encouraged to maintain good practices with the Flo-Tone device and inhaler-use monitor [14]. Patients requiring more urgent care will be assessed and treated by a rapid response team.

After the patients visit the MISSION Asthma clinic, the following tools will be used to enable continuous assessment, adjustment, and maintenance of disease control: Message Dynamics portal [15], Clinitouch system [16,17], myAsthma, Airsonett [18,19], smoking cessation, and education. Message Dynamics and Clinitouch use communications technology across multiple platforms to monitor symptoms, allowing early recognition of deterioration. By combining these approaches, HCPs can target patients early in an exacerbation event by using community-based management, thereby reducing the need for admission and the cumulative burden of symptoms on the patient. MyAsthma is a self-management online platform providing education and a symptom diary to encourage patients to manage their asthma. The Airsonett device is a laminar airflow device that reduces allergen exposure in patients with severe allergic asthma and may increase control and reduce healthcare utilization.

### Chronic Obstructive Pulmonary Disease

Patients will be identified through the use of GRASP COPD [13] and directed to the MISSION COPD clinics. At the clinics, patients will be reviewed according to the following the National Institute for Health and Care Excellence (NICE) quality framework standards [20]:

- Diagnosis: Thorasys [21] and exhaled nitric oxide from Niox [22]
- Medicine optimization: Flo-Tone device
- Smoking cessation
- Self-management: MISSION Self-Management Plans and myCOPD (similar to myAsthma but including online pulmonary rehabilitation program)

After the initial assessment, all patients will be encouraged and supported to keep themselves active while using myPR, the online pulmonary rehabilitation tool designed by my mHealth. Patients found to be at high risk of disease exacerbation (based on clinical characteristics or prior health care usage, decided by the senior clinician at the clinic) at the MISSION will be encouraged to report their symptoms via the Message Dynamics portal or Clinitouch system to allow early recognition and treatment of exacerbation.

### Breathlessness

#### Assessment

Patients with breathlessness who do not have an established diagnosis will be identified from the GP and practice records by using GRASP case-finding tools and will be offered the same high-quality diagnostic service as that offered to patients with asthma and COPD. In the MISSION clinic, these patients will be assessed using our novel diagnostic tools with additional availability of electrocardiogram recordings and a point-of-care test to identify any strain on the cardiac muscle by measuring the B-natriuretic peptide level. If asthma or COPD is identified, patients will join the respective care pathways. If a cardiac cause is identified, the patient will either be referred to the heart failure clinic or discharged to their GP. If the cause is a syndrome that leads to difficulty in breathing or deconditioning, patients will be offered an appointment with a MISSION ABC physiotherapist who will deliver targeted breathing retraining and discuss the benefits of maintaining exercise.

#### Innovations Embedded With New Models of Care

A brief outline of the innovations is provided below, followed by a description of how these innovations are incorporated into the new service model.

##### Digital Platforms to Identify and Monitor Patients

GRASP is a tool created by PRIMIS (Nottingham, United Kingdom) in conjunction with the University of Nottingham, United Kingdom. The tool examines GP records based on a chosen set of read codes and generates a search list tailored to the demands of the interrogator. This tool has been successfully used to generate patient lists for a variety of projects for the Wessex Academic Health Science Network (Southampton, United Kingdom).

##### Patient Self-Management and Monitoring

The following tools were used for patient self-management and monitoring:

###### MymHealth (myCOPD and myAsthma)

MymHealth [23] is a Web-based self-management system designed to support self-care in people with COPD and asthma. The system can be easily and securely accessed online by patients and HCPs. It facilitates the effective recognition of symptoms, inhaler technique, and management of medicines. It is an easy-to-use system that promotes patients to manage their COPD or asthma at home. Patients can also access an electronic platform containing a comprehensive, guided, 6-week pulmonary rehabilitation program to improve their health and quality of life.

###### Message Dynamics

Message dynamics [15] is a multiaward-winning provider of low-cost telehealth solutions through an app or a traditional telephone. Patients respond to a simple questionnaire that aims to detect symptoms of exacerbation.

###### Clinitouch

Clinitouch [16,17] is an app-based system that uses disease-control questionnaires and Bluetooth-enabled blood pressure and oxygen-saturation monitors that allow clinicians to remotely monitor their patient cohort. The patient is supported with an online self-management plan.

#### Technical Innovations

##### Diagnosis

The following tools were used for diagnosis:

###### Niox

This is a point-of-care testing device that measures exhaled nitric oxide [22]. It is a marker of eosinophilic corticosteroid-responsive inflammation that can predict loss of control in patients with asthma and supports phenotyping of patients with asthma and COPD. This tool was recently approved by NICE for the diagnosis of asthma [24].

###### Thorasys

This is a portable diagnostic tool that measures airways oscillometry, airway resistance, and obstruction independent of patient effort, thereby providing an accurate diagnosis with minimal effort [21,25]. Oscillometry offers an alternative method to traditional methods such as spirometry for assessing airway function. It has the potential to identify abnormal airway function that spirometry may be unable to detect, especially defects residing in the smaller airways. Oscillometry is particularly useful in patients who are unwilling or unable to adequately comply with the technical requirements of spirometry or when spirometry is considered inappropriate or contraindicated.

##### Treatment Improvement

For improving treatment, the following tools were used:

###### Clement-Clark Flo-Tone

This is a simple device that fits within the mouthpiece of any inhaler [26]. When the optimum inspiratory flow rate is achieved for that device, a musical note is heard. This immediately provides feedback to the patient but can also be recorded via a mobile phone app to inform the HCP.

###### Airsonett

The Airsonett device [19] can improve care of patients with severe allergic asthma and poorly controlled symptoms. The laminar airflow system, placed above the patient during sleep, vastly reduces aeroallergen exposure, resulting in fewer symptoms and improves disease control.

##### Service Model Innovations - MISSION COPD and MISSION Asthma

This is an award-winning, novel way of delivering highly specialized multidisciplinary asthma and COPD care in the community, providing specialist clinics to rapidly identify patients with asthma and COPD and then to assess and adjust management plans and empower patients through education [27]. A pathway has been designed to improve patient care and safety and prevent hospital admissions and nonelective visits in primary care.

### Aims and Objectives

This study aimed to examine (1) the implementation of a novel integrated care model for patients with airways disease and undifferentiated breathlessness using both quantitative and qualitative evaluation of processes and patient and HCP experiences and (2) clinical outcomes throughout the clinic cycles driving a continuous improvement process, evaluated through participatory action research. We also aimed to establish whether MISSION ABC, including innovative diagnostic and self-management tools, can deliver improvements in health service use and clinical outcomes for different patient groups (asthma, breathlessness, and COPD) by comparing the 12-month period prior to the first patient attendance and the 6-month period following attendance, by using regressional analyses to control for seasonal variation bias.

## Methods

### Study Design

#### Summary

The main focus of this project is to deliver a quality-improvement project—MISSION ABC—that has been informed by three prior projects. This protocol of predominantly Participatory Action Research [28] accompanies the project to record the delivery, iterative changes to the project, and outcomes and ensure rigor in the reporting of these parameters.

Accompanying innovations are embedded in the project to encourage a culture of growth amongst small- and medium-sized enterprises within the health care system. Outcomes related to these innovations are exploratory, not designed to be powered, and will not be presented with a control group.

A combination of study designs are required to evaluate all aspects of the service:

1. Participatory Action Research approach involving real-time evaluation at each clinic to inform subsequent clinics
2. Cohort (longitudinal) data approach for clinic- and patient-level service use and outcome indicators
3. Before-and-after study of patient outcomes before and after the clinic visit
4. Qualitative methods (interviews and focus groups)

#### Participatory Action Research Approach

##### Data Evaluation

A list of all the data that will be evaluated on a cyclical basis is provided below. These include quantitative data and themes that will be explored qualitatively with HCPs, patients, and carers to inform the next clinic. Issues for improvement, changes to the clinic process, and the rationale for changes will be recorded in a learning log. The impact of changes will be reviewed following the subsequent clinic visit and from any further feedback from qualitative interviews and focus groups. Fidelity to the original project plans and reasons for the changes made will be reported as part of the project analysis.

This list of outcomes is not exhaustive, as the cyclical approach may identify other key areas that are important to monitor. If additional questionnaires or methods of data collection from patients are required, they will be submitted to the ethics committee as an amendment.

##### Clinic Process

- Acceptability of clinic delivery model by patients, carers, and HCPs including host GP surgeries
- Perceived appropriateness of the clinic by the patient group, judged by HCPs delivering the clinic services and the host surgeries
- Primary care staff attendance at the clinic (number and position)
- Number of patients identified as appropriate for the clinic
- Attendance rates at each offered session

##### Diagnostics

- Number of new or modified diagnoses made at the clinic
- Number of patients where the British Thoracic Society Asthma stage or Global Obstructive Lung Disease (GOLD) COPD classification [5] is changed following discussion by the multidisciplinary team
- Number of new comorbidities newly identified

##### Education and Supported Self-Management

- Uptake of the education sessions when offered and reasons for decline, when given
- Acceptability of education sessions by participants and carers
- Confidence in self-management measured before and after the clinic visit and after subsequent education sessions
- Changes in treatment adherence before and after the intervention, measured using the Adherence Starts with Knowledge questionnaire-12 (ASK-12) [29,30] and a prescription reconciliation
- Uptake of the HCP education program
- Acceptability of the HCP education program by participants

##### Use of Treatment Tools

- Number of patients who showed improved inhaler technique, as judged after the first clinical encounter
- Number of patients with improved technique who sustained the improvement at subsequent review when prompted by clinical need
- Number of quit-for-life reviews that resulted in a quit attempt, and if these quit attempts resulted in sustained cessation at 6 months
- Number and type of inhalers prescribed before and after the intervention

##### Remote Monitoring

- Number of patients identified for remote monitoring
- Number of triggers on remote monitoring that progress to a clinically significant exacerbation
- Number of reported delays in reporting exacerbation due to the presence of remote monitoring
- Incidences where the carer of a family member is required to facilitate use of remote monitoring

##### Additional Balancing Measures

- Number of GP or practice nurse sessions changed or cancelled to host the clinic
- Additional costs such as childcare or extra travel incurred by the delivery team when care is delivered remote to their usual place of work or outside the usual working week
- Number of GP, community nurse, or emergency department episodes resulting from remote monitoring
- Number of secondary care referrals resulting from the program

#### Patient and Health Service Use Outcomes (Longitudinal Follow-Up)

Changes in quality-of-life measures prior to the clinic visits and at 3 and 6 months were measured with generic or disease-specific quality-of-life questionnaires. These questionnaires evaluated the following:

- Changes in productivity and activation measures prior to the clinic visit and at 6 months
- Changes in disease control, quality of life, and comorbidity measured by disease-specific questionnaires and unscheduled health care utilization (eg, emergency GP visits, out-of-hours/111 calls, hospital admissions, and emergency department attendances).
- Exacerbations in the 6 months before and after MISSION attendance
- Cost of delivery of the clinic model

#### Innovations

##### Thorasys

- Number of new diagnoses of airways disease made after use of the Thorasys device
- Number of patients who are unable to complete reproducible spirometry who have reproducible results with Thorasys
- Number of unusable results
- Perceived ease of use by health care professionals

##### Flo-Tone

- Number of patients given the Flo-Tone device
- Number of patients using the Flo-Tone device following reviews, when prompted by clinical need
- Number of patients deemed to have improved the pressurized metered dose inhaler technique through use of Flo-Tone

##### Airsonett

- Number of patients suitable for use of the Airsonett temperature-controlled laminar airflow device

- Number of patients offered the device who accepted its use
- Change in exacerbation frequency in the 6 months before and after the use of the Airsonett device
- Number of patients who elect for ongoing use of the device after 6 months

##### MyCOPD and MyAsthma

- Uptake of online, supported self-management provided by my mHealth
- Acceptability of online-supported self-management by the patient and family or carer
- Number of patients using online pulmonary rehabilitation
- Number of patients who access the online self-management plan

##### Message Dynamics

- Acceptability of the treatment by patient
- Acceptability of the treatment by HCPs
- Number of positive triggers that can be managed by advice only
- Number of positive triggers that require clinical review, and type of review chosen
- Number of triggers that result in a clinically significant exacerbation
- Duration of monitoring required by the patient, as judged by the clinical team

##### Clinitouch

- Acceptability of the treatment by patient

- Acceptability of the treatment by HCPs
- Number of positive triggers generated by Bluetooth device readings
- Number of positive triggers generated by symptom scores
- Number of positive triggers that can be managed by advice only
- Number of positive triggers that require clinical review, and type of review chosen
- Number of triggers that result in a clinically significant exacerbation
- Duration of monitoring required by the patient, as judged by the clinical team

### Study Participants

#### Study Setting

MISSION Clinics will be held in at least 10 surgeries within the Wessex region. The clinic services will be delivered by an integrated team of primary (primary care nurses and GPs), secondary (respiratory nurses, physiotherapists, physiologists, and registrars), and tertiary (regional specialist asthma service) care providers with delivery of care across all three sectors.

#### Overall Description of Study Participants

Adult patients (aged ≥ 16 years) with poorly controlled asthma or COPD as well as those with undifferentiated breathlessness will be identified using GRASP tools; referred from a community pharmacist or by their primary care team; and invited to a local MISSION clinic with a relative, friend, or carer in attendance, if they wish. All eligible attendees will be invited to participate in the study. The sample size will be determined by the uptake of appointments and clinic capacity but is projected to be 500 patients and 15 HCPs.

#### Eligibility Criteria

All patients who have attended the MISSION ABC clinic will be considered eligible to partake in the study if they are able to provide informed consent. Family or carers will be asked to participate in the qualitative research if they have accompanied the patient to an educational event. Health care participants from host surgeries or visiting from outside organizations will also be eligible for participation in the participatory action research and qualitative aspects of the study. An individual’s participation in any applicable aspect of the MISSION will be unaffected by their decision to consent.

### Study Procedures

#### Screening and Enrollment

All potential participants will be sent a Participant Information Sheet by post or email after initial contact. If they are suitable for the clinic, they will be screened for their ability to give informed consent. If they are able to consent and wish to do so, they will be enrolled at a clinic. It will be made clear to each participant that their care is unaffected by their decision to enroll in the study.

#### Randomization

There will be no randomization.

#### Study Assessments

The study assessments are summarized in Figures 1, 2, and 3 (COPD, asthma, and breathlessness, respectively).

#### Patient Assessments

##### Patient Characteristics

- Age, height, weight, and body mass index recorded at the first clinic visit
- New or changed diagnoses recorded for each patient seen
- Any change in British Thoracic Society Asthma Stage or GOLD COPD classification, as judged by the multidisciplinary team for those with newly diagnosed or established asthma or COPD
- Number and type of comorbidities pre-existing and identified through the MISSION process
- Occupation, employment status, and postal code
- Smoking status

##### Disease Control, Disease-Related Quality of Life, and Activation Measures

All baseline questionnaires will be sent to patients before the first clinical encounter. If they choose not to participate in the research study, these questionnaires will still aid in clinical decision-making and will be retained in the clinical record. If patients need assistance in completing these questionnaires, it will be offered at the first clinic visit. The questionnaires used are listed below:

- A baseline exploratory semistructured questionnaire designed by the team to explore disease impact and behaviors
- Disease-specific control questionnaires (Asthma Control Questionnaire [31] and COPD Assessment Test [32]): Baseline measure and then repeated at 3 and 6 months after the first clinical encounter
- Exercise tolerance and symptom measures: Veterans Specific Activity Questionnaire [33], Nijmegen questionnaire [34], and Self Evaluation of Breathing Questionnaire [35] at baseline and repeated at 3 and 6 months
- Use of unscheduled care (including GP visits), steroids or antibiotics for exacerbations, and hospital admissions recorded for each patient at 3 and 6 months compared to their 6 and 12 months visits prior to the MISSION process
- Measures of activation and medicine compliance (ASK-12 and Patient Activation Measure [36]) at baseline and 3 and 6 months
- Productivity measures (EuroQoL-5D [37] and Work Productivity and Activity Impairment [38]) at baseline and 6 months
- Quality of life scores: Short Form Health Survey-36 (generic) [39], Asthma Quality of Life Questionnaire (AQLQ) for asthma [40], and St George’s Respiratory Questionnaire for COPD [41]
- Number of quit-for-life reviews that have resulted in a quit attempt at 3 and 6 months
- Prescription reconciliation at 6 months before and after the clinic visit to assess medication usage

##### Patient’s Experience of MISSION ABC

- Semistructured questionnaires exploring patient experiences, completed after each clinical encounter
- Qualitative interviews will be completed with 10% of patients who participate in the study, exploring factual, structural, interpersonal, intrapersonal, and contextual influences on the experience of receiving the new service. Discussions will be transcribed using a transcription service and analyzed using a thematic analysis to compare themes. A second researcher will review the analysis to ensure all themes are captured.

#### Assessment of Innovations

##### Devices

- Acceptability of use of the new diagnostic devices (Niox, FeNO, and Thorasys) as well as the management tools (Flo-Tone and Airsonett) will be analyzed through structured questionnaires given to the clinic staff.
- Instances where a diagnosis is made as a result of the use of these devices, which would not be identified by standard investigation, will be recorded. If these events are unclear, the decision of the senior clinician present will be considered.
- Equipment breakdowns or technical difficulties will be recorded as a clinic process outcome.
- Proportion of patients who have continued to use supportive devices at the follow-up review will be calculated.

##### Digital Platforms

- HCPs’ and patients’ experiences of the use of digital platforms will be assessed using semistructured questionnaires.

#### Assessment of Monitored, Supported Follow-Up

- Number of patients offered remote monitoring, and proportion of patients who accept
- Proportion of patients with access to smartphones or home internet
- Number of triggers generated on remote monitoring
- Proportion of triggers that result in a clinically significant exacerbation
- Proportion of triggers that require a patient review
- Proportion of clinically significant exacerbations that are managed in the community
- Proportion of responses to remote monitoring that are completed by the patient
- Rate of drop outs from remote monitoring and the stated reason
- Patient and primary care experiences of using remote monitoring measured by a semistructured questionnaire

#### Assessment of Health Care Professionals’ Experiences

In addition to the assessments listed above, a sample of 10 HCPs will be invited to participate in a focus group that will explore their views on the acceptability, appropriateness, and feasibility of the program. An independent interviewer will ask them to comment on their perceived barriers and drivers for further implementation of the program. The focus group will be recorded, but the responses will be anonymized. After transcription, the interviews will be analyzed for themes.

#### Participatory Action Research Outcomes

The core team will meet monthly to evaluate the clinic delivery process. Using patient and HCP feedback questionnaires, pitfalls and issues of the clinical process will be examined using Plan-Do-Study-Act analysis. When changes are made, a further review will take place in the following meeting until the change is deemed to have provided a positive impact on the process. These analyses will be recorded in a learning log.

#### Additional Process Outcomes

- Rate of uptake of the clinic by primary care providers when offered
- Expenditure in delivery of the clinic services including staffing costs, consumables, and unexpected expenses

#### Balancing Measures

We acknowledge that enhanced investigation and intervention may increase costs to health care providers in the short-to-medium term. The following balancing measures will be included as a project outcome:

- Number of GP or practice nurse sessions changed or cancelled to host the clinic
- Additional costs such as childcare or extra travel incurred by the delivery team when care is delivered remote to their usual place of work or outside the usual working week
- Number of additional GP, community nurse, or emergency department episodes resulting from remote monitoring
- Number of additional secondary care referrals resulting from the program

#### Discontinuation or Withdrawal of Participants from Study Treatment

Participants may withdraw at any point in the study.

#### Definition of End of Study

The end of study is the 6-month follow-up after the questionnaires are received from the last participant.

### Data Analysis

#### Description of Analysis Populations

All patients recruited in the study will be included in the analysis population.

#### Analysis of Quantitative Outcome Data

The objective of all analyses is to examine differences in measured variables between time points.

The following comparisons will be made:

- Asthma Control Questionnaire, CAT, and AQLQ: baseline as compared to 3 and 6 months
- Emergency care outcomes: 3 and 6 months prior to study as compared to the 3- and 6-month study period
- Activation or medicine compliance (ASK-12 and Patient Activation Measure): baseline as compared to 3 and 6 months
- EuroQoL-5D and weight-bearing physical activity: baseline as compared to 3 and 6 months

All the abovementioned variables are continuous measures. Comparisons between time points will be made using the paired *t* test or the Wilcoxon matched-pairs test, depending on the distribution of the changes in outcome between time points.

Summaries of the measures of monitoring and supported follow-up will be prepared (measures outlined above). Patient satisfaction measures will be summarized descriptively. Numbers and percentages will be used for categorical variables, whereas mean and SD or median and interquartile range will be used for continuous variables. Uncertainty in percentages, means, and medians will be quantified by calculating the appropriate 95% CIs.

#### Participatory Action Research

Periodic reviews of process measures and feedback may result in iterative changes of the clinical model. The fidelity of clinic delivery to the original plan will be presented and analyzed.

#### Procedure for Dealing With Missing, Unused, and Spurious Data

Each outcome will be analyzed using measured data values of all patients. Patients with missing data will be excluded from the analyses. Suspicious data values will be checked against the source data. If there are outlying values, the analyses will be performed twice—once with the outlying values and once without the outlying values.

#### Interim Analysis and Criteria for Early Study Termination

No interim analyses are planned. A single analysis will be performed when all patients have completed the study. The study will not be terminated early based on any study data.

### Patient Public Involvement

#### Study Design

The MISSION ABC clinic has been formed by feedback from patients’ involvement in the prior pilots (MISSION Asthma and MISSION COPD). All relevant outcomes of patients will be recorded. All paperwork, such as Participant Information Sheets and educational literature, is reviewed by our patient participants before distribution.

#### Study Implementation

The steering group for MISSION ABC includes the chair of a local Patient Action Group and patients from the MISSION COPD pilot project and local Breathe Easy Groups. Ongoing data such as those generated from the cyclical evaluation of clinics will be reviewed, and the group will contribute to informing and delivering change.

#### Dissemination

Lay summaries of the results of the research will be written together with the Patient and Public Involvement members, disseminated both in verbal and written formats to local groups such as Breathe Easy groups, and written in appropriate formats for the Trust social media accounts. The Patient and Public Involvement members will be invited to present data locally and nationally, where appropriate.

## Results

The project was funded in 2017 and enrollment was completed in 2018. Data analysis is currently underway, and the first results are expected to be submitted for publication in April 2019.

## Discussion

This is a unique participatory action research study using both qualitative and qualitative methodology involving patients, carers, and HCPs. The longer-term impact of the service will be evaluated using clinical and health service outcomes that will inform decisions on future service implementation.

## Abbreviations

ASK-12: Adherence Starts with Knowledge questionnaire-12
AQLQ: Asthma Quality of Life Questionnaire
COPD: chronic obstructive pulmonary disease
FeNO: fractional exhaled nitric oxide
GP: general practitioner
GOLD: Global Obstructive Lung Disease
GRASP: Guidance on Risk Assessment in Stroke Prevention
HCP: health care professional
MISSION ABC: Modern Innovative Solution Improving Outcomes in Asthma, Breathlessness, and COPD
NICE: National Institute for Health and Care Excellence
